# The “Eclectic System” of Obstetrics

**Published:** 1874-07

**Authors:** C. C. Hart

**Affiliations:** Cross Keys, Ga.


### THE "ECLECTIC SYSTEM" OF OBSTETRICS.

By C. C. HART, M.D., of Cross Keys, Ga.

Mrs. P., aged nineteen, primipara, well developed and very stout, gave birth to a living male child, in a sitting position upon the lap of her husband, under the direction and administration of a, so-called, *eclectic doctor* of the neighborhood. After the child was delivered the doctor, so-called, put the patient to bed, and instituted an examination to see if all had gone well. Finding a mass occupying the vagina, which he diagnosticated to be prolapsus uteri, he proceeded to plug the vagina with cotton to elevate the mass to what he esteemed to be its proper position in the body. He visited his patient daily, steaming her up industriously with, so-called, composition tea "to sweat her and keep down fever." In this purpose, however, he signally failed, for on April 13th I was requested by Mr. P. to visit his wife, who was suffering great pain, with abdominal tenderness and high febrile reaction. Shortly before my arrival Mrs. W., a neighboring lady of good common sense, had removed from the vagina the cotton plug and a large putrid mass, which she supposed to be the decomposed placenta, and which she had placed in the fire. She exhibited, however, in the vessel a quantity of disintegrated blood clot and lochia, which had been retained for six days by the tampon, until it had become excessively fœtid.

Under active detergent and disinfectant treatment the patient ultimately made a good recovery, thanks to that marvel worker, *nature.* What, likely, would have been the result had not the good sense of Mrs. W., or some body else, come to the rescue?

NOTE.—Years ago a very clever gentleman, (clever in the American, not the English, sense), of the, so-called, eclectic school attended his own wife in a first labor. All seemed to move on well for the time, but, on the sixth or seventh day, her condition being unsatisfactory, he asked a neighboring physician, of character and skill, to see her. The latter observing a very unusual stench about the puerperal bed, inquired: "Did the placenta come away all right and complete?" Eclectic replied, with exceeding *naivete:* "The child came all right and perfect, but I don't know what you mean by *placenta,* doctor."—ED.

